# Observational study of HR+/HER2− metastatic breast cancer patients treated with abemaciclib in Spain in the Named Patient Use Program (AbemusS)

**DOI:** 10.1007/s12094-023-03159-9

**Published:** 2023-04-07

**Authors:** Salvador Blanch, Juan Miguel Gil-Gil, Miriam Arumí, Elena Aguirre, Miguel Ángel Seguí, Manuel Atienza, Silvia Díaz-Cerezo, Alberto Molero, José Manuel Cervera, Joaquín Gavilá

**Affiliations:** 1 Instituto Valenciano de Oncología (IVO), Carrer del Professor Beltrán Báguena, 8, 46009 Valencia, Valencia Spain; 2 Institut Català d’Oncologia (ICO), Gran Via 199-201, Hospitalet, 08908 Barcelona, Spain; 3 Department of Medical Oncology, Vall d’Hebron University Hospital and Vall d’Hebron Institute of Oncology, Passeig de la Vall d’Hebron, 119, 08035 Barcelona, Spain; 4 Hospital Universitario Miguel Servet, Paseo Isabel la Católica, 1-3, 50009 Zaragoza, Spain; 5 Hospital Parc Taulí de Sabadell, Parc Taulí, 1, 08208 Sabadell, Barcelona Spain; 6 Medical Department, Lilly Spain, Av. de la Industria 30, 28108 Madrid, Spain

**Keywords:** Metastatic breast cancer, Abemaciclib, Effectiveness, Real world, HR+/HER2− Spain

## Abstract

**Introduction/objectives:**

To describe abemaciclib use in patients with hormone receptor-positive, human epidermal growth factor receptor-negative (HR+/HER2−) metastatic breast cancer (mBC) who participated in the Named Patient Use program (NPU) in Spain.

**Material and methods:**

This retrospective study was based on medical record review of patients across 20 centers during 2018/2019. Patients were followed up until death, enrolment in a clinical trial, loss of follow-up or study end. Clinical and demographic characteristics, treatment patterns and abemaciclib effectiveness were analyzed; time-to-event and median times were estimated using the Kaplan–Meier (KM) method.

**Results:**

The study included 69 female patients with mBC (mean age 60.4 ± 12.4 years), 86% of whom had an initial diagnosis of early BC and 20% had an ECOG ≥ 2. Median follow-up was 23 months (range 16–28). Metastases were frequently observed in bone (79%) and visceral tissue (65%), with 47% having metastases in > 2 sites. Median number of treatment lines before abemaciclib was 6 (range 1–10). Abemaciclib monotherapy was received by 72% of patients and combination therapy with endocrine therapy by 28% of patients; 54% of patients required dose adjustments, with a median time to first adjustment of 1.8 months. Abemaciclib was discontinued in 86% of patients after a median of 7.7 months (13.2 months for combination therapy and 7.0 months for monotherapy) mainly due to disease progression (69%).

**Conclusion:**

These results suggest that abemaciclib is effective, as monotherapy and in combination, for patients with heavily pretreated mBC, consistent with clinical trial results.

## Introduction

Breast cancer (BC) is the most frequent cancer among women in Spain, with an estimated 35,000 new cases in 2021 [1]. Despite recent improvements in early detection and treatment outcomes, 30% of patients with early-stage BC present with metastatic breast cancer (mBC) during follow-up [2]. In the last decade, median survival for mBC was around 39.5 months, with an observed 5-year survival rate of 33.8% [3].

BC has different biological subtypes depending on expression of the estrogen receptor (ER), the progesterone receptor (PR) and the human epidermal growth factor receptor (HER2). HR+/HER2− breast cancer, representing approximately 70% of all subtypes [5], has a better prognosis and survival with lower risk of metastasis [6], but presents a particular pattern of metastasis, with a higher rate of bone metastasis than other subtypes [7]. Endocrine therapy (ET) has long been the recommended first-line option for HR+/HER2− mBC [2]. Nevertheless, most patients show resistance to ET, requiring consecutive alternative targeted therapy or combinations with ET followed by chemotherapy [2]. However, a reduction in effectiveness occurs between first and subsequent lines of ET and chemotherapy, despite major advances with new treatments [4]. Therefore, a substantial unmet medical need remains in HR+/HER2− mBC.

In recent years, several cyclin-dependent kinase (CDK) inhibitors have been approved for HR+/HER2− mBC [8]. CDK4/6 inhibitor (CDK4/6i) therapies in combination with ET have shown improved progression-free survival (PFS) compared with ET alone [9]. In 2018, a new CDK4/6i, abemaciclib, was approved for the treatment of women with HR+/HER2− locally advanced or mBC in combination with an aromatase inhibitor or fulvestrant as initial endocrine-based therapy, or in women who have received prior ET, by the European Medicines Agency (EMA) [10], based on the results of MONARCH 2 [11] and MONARCH 3 [12] studies. These clinical trials demonstrated that PFS and objective response rate (ORR) were significantly higher in patients treated with abemaciclib + ET than in those who received fulvestrant or non-steroidal aromatase inhibitors (NSAIs) alone, respectively. In addition, abemaciclib was approved as monotherapy in 2017 by the US Food and Drug Administration (FDA) for HR+/HER2− mBC with disease progression following ET and prior chemotherapy [13], based on the results of the Phase II clinical trial MONARCH 1 [14].

After EMA approval and before abemaciclib was commercially available in Spain, a Named Patient Use (NPU) program for abemaciclib was authorized, running from July 2018 to April 2019. The NPU allowed inclusion of patients with advanced or metastatic HR+/HER2− BC, who received abemaciclib free of charge. Abemaciclib could be prescribed as monotherapy or in combination with fulvestrant or a NSAI at the physician´s discretion. A total of 98 patients from 39 centers across Spain were included. Given the limited real-world data available on treatment patterns and outcomes in patients with HR+/HER2− mBC treated with abemaciclib, data from routine clinical practice is needed.

We describe here the findings of the AbemusS study, conducted to obtain real-world data on patient characteristics, treatment patterns and abemaciclib effectiveness in patients with HR+/HER2− mBC from the NPU program in Spain.

## Methods

### Study design and objectives

AbemusS was a retrospective observational study based on hospital medical records with a primary objective to describe patient characteristics and treatment patterns for patients with HR+/HER2− advanced or mBC who initiated abemaciclib treatment within the NPU program in Spain, between July 1, 2018 and April 30, 2019. Patients eligible for the NPU program were those with a diagnosis of advanced or metastatic HR+/HER2− BC, a recent laboratory assessment and who were not candidates for any therapeutic alternative (except chemotherapy) in Spain (including other available CDK4/6 inhibitors or clinical trials), as confirmed by their physician. Within the NPU program, the patients could receive abemaciclib as monotherapy or in combination with fulvestrant or an aromatase inhibitor. In addition, to participate in the study, patients had to be female and aged ≥ 18 years at the time of inclusion. There were no other specific exclusion criteria.

The 20 centers with the highest patient numbers included in the NPU program were selected. All qualifying patients from each site were included in the study.

The index date was defined as the start of abemaciclib treatment, and patients were followed until death, enrollment in a clinical trial, loss to follow-up or end of the study period (December 31, 2020), whichever was earliest. A retrospective review of medical records of two periods was performed: the pre-index period (from first BC diagnosis to the start of abemaciclib) and the post-index period (from the index date to the end of follow-up) (Appendix, Figure 4).

**Fig. 1:**
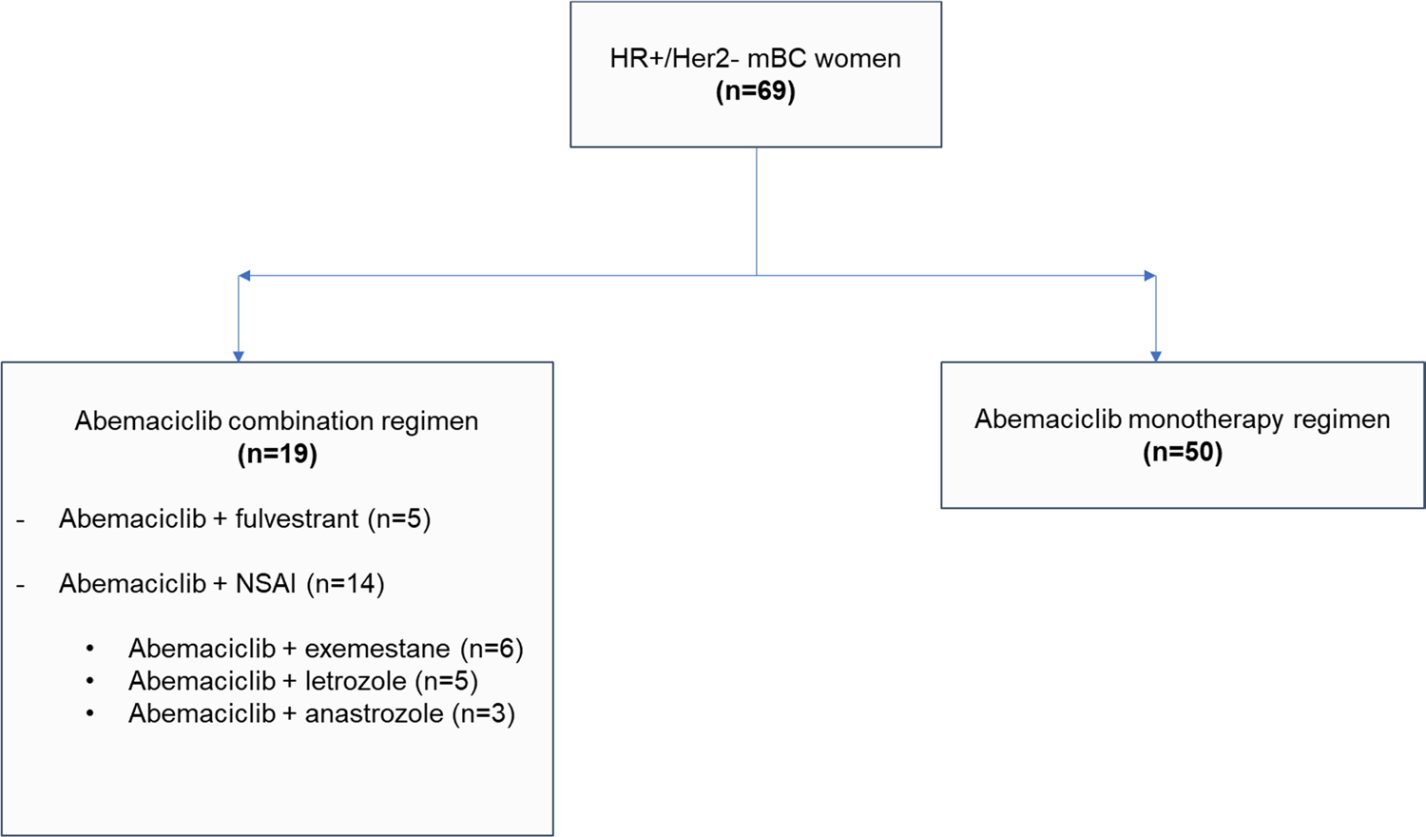
AbemusS study population. *mBC* metastatic breast cancer, *NSAI* non-steroidal aromatase inhibitor

### Study variables

At the index date, patient age, smoking status, body mass index (BMI), location and number of metastatic lesions, Eastern Cooperative Oncology Group (ECOG) performance status [15], disease measurability, histological grade, HR status, Ki67 (%), abemaciclib regimen and dosing schedule were recorded. For the pre-index period, medical history and breast cancer variables (initial stage of BC at diagnosis, date of diagnosis, neoadjuvant treatment, surgery, adjuvant treatment, number of lines of treatment in mBC and treatments within each line) were collected.

For the post-index period, information pertaining to dose adjustments during the follow-up period, abemaciclib discontinuation, reasons for discontinuation (information on specific adverse events was not obtained), number of treatment lines after abemaciclib and treatments within each line, best treatment response (complete response (CR), partial response (PR), stable disease, progressive disease (PD) defined by RECIST 1.1 criteria [16]), date of last contact and reasons for end of follow-up was obtained.

### Statistical analysis

A descriptive analysis of all variables was performed. Continuous variables were described by number of patients with valid/missing observations, mean, standard deviation (SD), median, 25th and 75th percentiles (P25-P75), and minimum and maximum values. Categorical variables were described by frequencies and related percentages. A p-value lower than 0.05 was considered significant. Time-to-event rates at 6, 12 months and end of follow-up were estimated using Kaplan–Meier curves.

Patients were grouped according to the setting of their resistance to ET (adjuvant (eBC) or metastatic (mBC)) and whether it was primary or secondary based on ESMO criteria [17].

Outcomes used to describe treatment patterns were: abemaciclib time on treatment, the proportion of patients who discontinued during follow-up, the proportion of patients requiring dose adjustment and time to first dose adjustment.

To measure the secondary objective of abemaciclib effectiveness, the following variables were calculated: overall survival (OS) and PFS (median and rates at 6, 12 months and at end of follow-up), time to progression (TTP), ORR, time to response (TTR), duration of response (DoR), disease control (DC) and clinical benefit rate (CBR).

All analyses were conducted separately for monotherapy and combination therapy groups.

Due to the observational nature of this study, and since no hypothesis testing was attempted, a formal calculation of sample size and statistical power was not applicable. However, for informative purposes only, we determined that a sample size of approximately 71 patients was needed to allow estimation of dichotomic variables with a precision of 0.05 to 0.1 and a precision of 0.10 or 0.15 for continuous variables with a confidence level of 95%. All analyses were conducted using SAS version 7.15 Enterprise Guide.

## Results

### AbemusS study population

The 20 participating sites added a total of 71 patients enrolled in the NPU program, however 2 patients never received abemaciclib and were excluded from the study. The AbemusS study population comprised 69 evaluable patients. Nineteen (27.5%) patients initiated abemaciclib as part of a combination regimen, 5 with fulvestrant and 14 with an NSAI (6 exemestane, 5 letrozole and 3 anastrozole), and 50 patients (72.5%) received abemaciclib monotherapy (Fig. 1). Median follow-up was 23.4 months for monotherapy and 22.5 months for combination treatment.

### Patient demographic and clinical characteristics

Abemaciclib was started at a mean (SD) age of 62.1 (11.2) years in the monotherapy group and 55.8 (14.3) years in the combination therapy group, with 30.0% and 10.5% of patients > 70 years old, respectively. ECOG status of 1 was reported in 54.1% of patients receiving monotherapy and 53.8% of patients receiving combination therapy, with an ECOG status of 2 in 21.6% and 7.7% of patients, respectively. At the start of treatment, 75.5% of patients in the monotherapy and 73.6% of patients in the combination therapy group had ≥ 2 metastatic sites, with bone (87.8% and 57.9%) and visceral (61.2% and 73.7%) the most frequent metastatic sites. In the monotherapy group, 13.8% of visceral metastasis were central nervous system metastases, with a corresponding value of 21.4% for the combination therapy group (Table 1).

**Table 1:** Patients’ characteristics at the index visit

	Monotherapy (n = 50)	Combination therapy (n = 19)
Age at index		
Mean (SD)	62.1 (11.2)	55.8 (14.3)
BMI (kg/m^2^) at treatment start with abemaciclib		
Mean (SD)	24.9 (3.9)	28.0 (6.9)
Median	25.3	27.5
N missing	20	6
BMI cat		
Underweight: BMI < 18.5 kg/m^2^	2 (6.7%)	0
Normal weight: 18.5 kg/m^2^ ≤ BMI ≤ 25 kg/m^2^	11 (36.7%)	4 (30.8%)
Overweight: 25 kg/m^2^ < BMI ≤ 30 kg/m^2^	16 (53.3%)	6 (46.2%)
Obese: 30 kg/m^2^ < BMI ≤ 35 kg/m^2^	1 (3.3%)	1 (7.7%)
Severely obese: BMI > 35 kg/m^2^	0	2 (15.4%)
N missing	20	6
Smoking status		
Current smoker	2 (6.3%)	4 (26.7%)
Ex-smoker	6 (18.8%)	3 (20.0%)
Non-smoker	24 (75.0%)	8 (53.3%)
N missing	18	4
ECOG		
0	8 (21.6%)	5 (38.5%)
1	20 (54.1%)	7 (53.8%)
2	8 (21.6%)	1 (7.7%)
3	1 (2.7%)	0
4	0	0
5	0	0
N missing	13	6
Number of metastatic sites		
Mean (SD)	2.6 (1.3)	2.3 (1.1)
Median	3.0	2.0
N missing	1	0
Number of metastatic sites (categorical)		
1	12 (24.5%)	5 (26.3%)
Visceral only	2 (4.1%)	2 (10.5%)
Bone only	10 (20.4%)	2 (10.5%)
Lymph nodes only	0	0
Soft tissue only	0	0
Skin only	0	0
Breast only	0	0
Other only	0	1 (5.3%)
2	12 (24.5%)	7 (36.8%)
> 2	25 (51.0%)	7 (36.8%)
N missing	1	0
Location of metastatic sites		
Visceral	30 (61.2%)	14 (73.7%)
Lung	15 (51.7%)	5 (35.7%)
Liver	22 (75.9%)	11 (78.6%)
Central nervous system	4 (13.8%)	3 (21.4%)
Valid n	29	14
N missing	1	0
Bone	43 (87.8%)	11 (57.9%)
Lymph nodes	12 (24.5%)	7 (36.8%)
Soft tissue	6 (12.2%)	0
Skin	0	3 (15.8%)
Breast	2 (4.1%)	0
Other	15 (30.6%)	4 (21.1%)
N missing	1	0
Disease measurability		
Non-measurable	12 (26.1%)	4 (21.1%)
Measurable	34 (73.9%)	15 (78.9%)
N missing	4	0
Grade of differentiation		
Well differentiated	6 (19.4%)	1 (10.0%)
Moderately differentiated	17 (54.8%)	6 (60.0%)
Poorly differentiated	8 (25.8%)	3 (30.0%)
N missing	19	9
Ki67		
Mean (SD)	32.7 (21.9)	21.3 (11.1)
Median	40.0	25.0
Progesterone receptor (PgR)		
−	6 (16.2%)	8 (53.3%)
+	31 (83.8%)	7 (46.7%)
Valid n	37	15
Performed PgR but unknow status	2	0

*ECOG* Eastern Cooperative Oncology Group, *BC* breast cancer, *SD* standard deviation

About 84.0% of patients receiving monotherapy and 89.5% receiving combination therapy had an initial breast cancer diagnosis as early-stage disease, with a median time from diagnosis to initiation of abemaciclib of 15.3 years and 11.2 years, respectively.

### Treatment patterns (previous treatments and abemaciclib)

Median time from mBC diagnosis to initiation of abemaciclib was 7.0 and 4.9 years in the monotherapy and combination therapy groups, respectively.

In the monotherapy group, the median number of prior lines in the mBC setting was 6.0, while in combination with ET, patients had a median of 5.0 lines. All patients in each group received ≥ 1 prior treatment (Table 2); 94.0% of patients in the monotherapy group and all patients in the combination therapy group had received prior ET. ET was administered as first-line treatment in 72.0% and 68.4% of patients in the monotherapy and combination therapy groups, respectively; 66.0% and 66.7% as second line; and 54.2% and 41.2% as third line. No patients received any prior targeted therapies in neither group. In the monotherapy group, 17.0% of patients presented with primary endocrine resistance (87.5% adjuvant, 12.5% metastatic) and 57.4% with secondary resistance (77.8% adjuvant, 22.2% metastatic). In the combination therapy group, primary endocrine resistance was observed in 21.1% of patients (75.0% adjuvant, 25.0% metastatic), while secondary endocrine resistance was observed in 26.3% (80.0% adjuvant, 20.0% metastatic) (Table 3).

**Table 2:** Metastatic BC treatment history

	Monotherapy (n = 50)	Combination therapy (n = 19)
Number of treatment lines (metastatic BC) prior to abemaciclib treatment		
Mean (SD)	6.38 (2.12)	5.42 (2.27)
Median (P25–P75)	6.0 (5.0–8.0)	5.0 (4.0–7.0)
(Min; max)	(2.0; 10.0)	(1.0; 9.0)
Valid n	50	19
N missing	0	0
Number of lines (metastatic BC) - categorical		
1	0	1 (5.3%)
2	2 (4.0%)	1 (5.3%)
3	2 (4.0%)	2 (10.5%)
4	4 (8.0%)	2 (10.5%)
5	11 (22.0%)	4 (21.1%)
6	9 (18.0%)	3 (15.8%)
7	7 (14.0%)	2 (10.5%)
8	4 (8.0%)	2 (10.5%)
9	7 (14.0%)	2 (10.5%)
10	4 (8.0%)	0
Valid n	50	19
N missing	0	0

*BC* breast cancer, *P25–P75* median 25 and 75 percentiles, *SD* standard deviation

**Table 3:** Classification of endocrine resistance

	Monotherapy	Combination therapy
Endocrine therapy		
No	3 (6.0%)	0
Yes	47 (94.0%)	19 (100.0%)
Valid n	50	19
Resistance to endocrine therapy		
Primary endocrine resistance^a^	8 (17.0%)	4 (21.1%)
Adjuvant	7 (87.5%)	3 (75.0%)
Metastatic	1 (12.5%)	1 (25.0%)
Secondary endocrine resistance^b^	27 (57.4%)	5 (26.3%)
Adjuvant	21 (77.8%)	4 (80.0%)
Metastatic	6 (22.2%)	1 (20.0%)
Without endocrine resistance	11 (23.4%)	9 (47.4%)
Insufficient information	1 (2.1%)	1 (5.3%)
Valid n	47	19
N missing	0	0

^a^Recurrence on the first two years while on adjuvant endocrine therapy (ET) in adjuvant setting or PD within the first 6 months while on first line ET in metastatic setting

^b^Recurrence while on adjuvant ET but after the first 2 years, or recurrence within 12 months of completing adjuvant ET, in adjuvant setting or PD ≥ 6 months after initiating ET or while ET in metastatic setting (17)

The median duration of abemaciclib treatment was 7.0 months in the monotherapy group, and 13.2 months in the combination therapy group. In the monotherapy group, 52.0% of patients received abemaciclib at a dose of 150 mg (a mean [SD] number of cycles of 8.1 [7.0]) and 48% received the 200 mg dose (3.6 [5.8]). For patients in the combination therapy group, all received abemaciclib 150 mg with a mean (SD) of 11.7 (6.9) cycles, with 73.7% receiving an NSAI and 26.3% fulvestrant. A total of 52.0% of patients in the monotherapy group and 57.9% in the combination therapy group needed dose adjustments, with median time to first dose adjustment of 1.9 and 1.7 months, respectively. At the end of follow-up, abemaciclib had been discontinued in 86.0% of patients in the monotherapy group and 84.2% in the combination therapy group. The main reason for discontinuation was PD in both groups, observed in 67.4% and 75.0% of patients, respectively. The probability of continuing abemaciclib at six months was 54.0% for patients receiving monotherapy and 68.4% for combination therapy. After abemaciclib treatment, 62.0% of patients receiving monotherapy and 63.1% receiving combination therapy received a mean (SD) of 1.3 (1.3) and 1.1 (1.2) lines of treatment, respectively. Chemotherapy was the most frequent first-line treatment after abemaciclib, received by 90.3% and 75.0% of patients in the monotherapy and combination therapy groups, respectively, with chemotherapy received as second and third-line therapy in 78.9% and 75.0%, and in 75.0% and 100.0% of patients, respectively.

### Abemaciclib effectiveness

A CR was achieved in 2.3% of patients receiving abemaciclib monotherapy and 11.8% of patients receiving combination therapy, with a PR in 23.3% and 23.5%, respectively. ORR at the end of follow-up was 25.6% and 35.3% for the monotherapy and combination therapy groups, respectively. Median TTR was estimated at 5.0 months and 3.0 months for the monotherapy and combination therapy groups, respectively, with a median DoR of 5.7 months for the monotherapy group (not available for combination therapy group). DC was achieved at the end of follow-up in 65.1% of patients receiving abemaciclib monotherapy and 70.6% receiving combination therapy, with a CBR at 6 months of 60.7% and 58.3%, respectively, and at 12 months of 46.4% and 41.7%, respectively (Table 4).

**Table 4:** Abemaciclib effectiveness

	Monotherapy	Combination therapy
Tumor response evaluated by imaging		
No	7 (14.0%)	2 (10.5%)
Yes	43 (86.0%)	17 (89.5%)
Valid N	50	19
RECIST criteria		
Complete response (CR)	1 (2.3%)	2 (11.8%)
Partial response (PR)	10 (23.3%)	4 (23.5%)
Stable disease	17 (39.5%)	6 (35.3%)
Progressive disease	15 (34.9%)	5 (29.4%)
Valid n	43	17
N missing	0	0
DC		
At end of follow-up or death	28 (65.1%)	12 (70.6%)
Valid N	43	17
CBR		
At 6 months	17 (60.7%)	7 (58.3%)
At 12 months	13 (46.4%)	5 (41.7%)
Valid N	28	12
ORR		
At end of follow-up or death	11 (25.6%)	6 (35.3%)
Valid N	43	17

CBR: clinical benefit rate was calculated as the proportion of patients with SD, CR or PR who had stable disease or whose tumor had shrunk at 6 and 12 months

DC: disease control calculated as the proportion of patients who had CR, PR or stable disease after index date (only with response evaluated by imaging)

ORR: objective response rate calculated as the proportion of patients with CR and PR divided by the total of patients with tumor response by imaging and with information of RECIST criteria

**Fig. 2:**
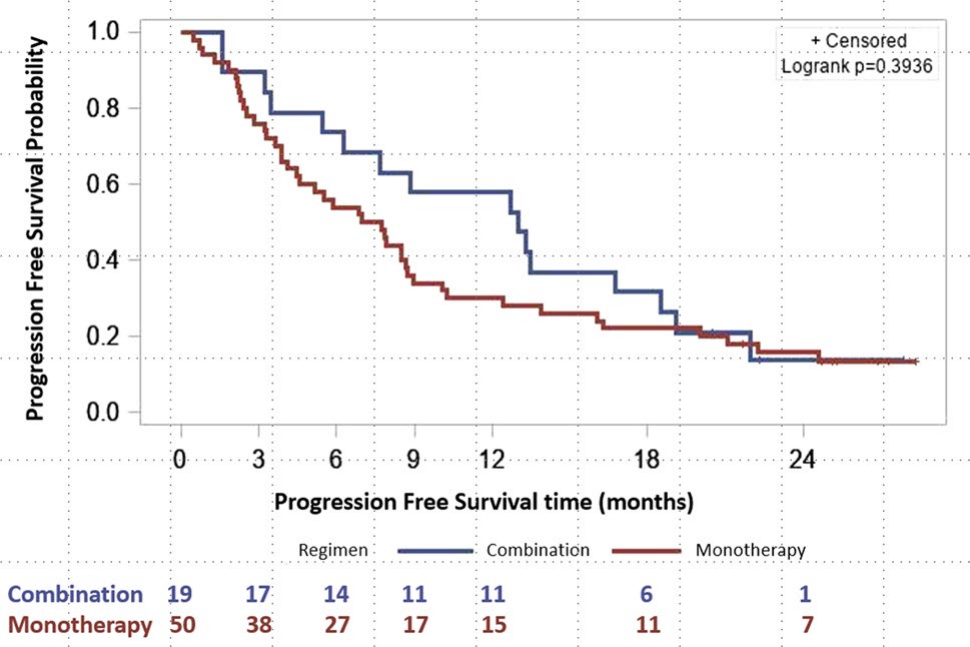
Progression-free survival according to regimen received (abemaciclib as monotherapy or in combination)

**Fig. 3:**
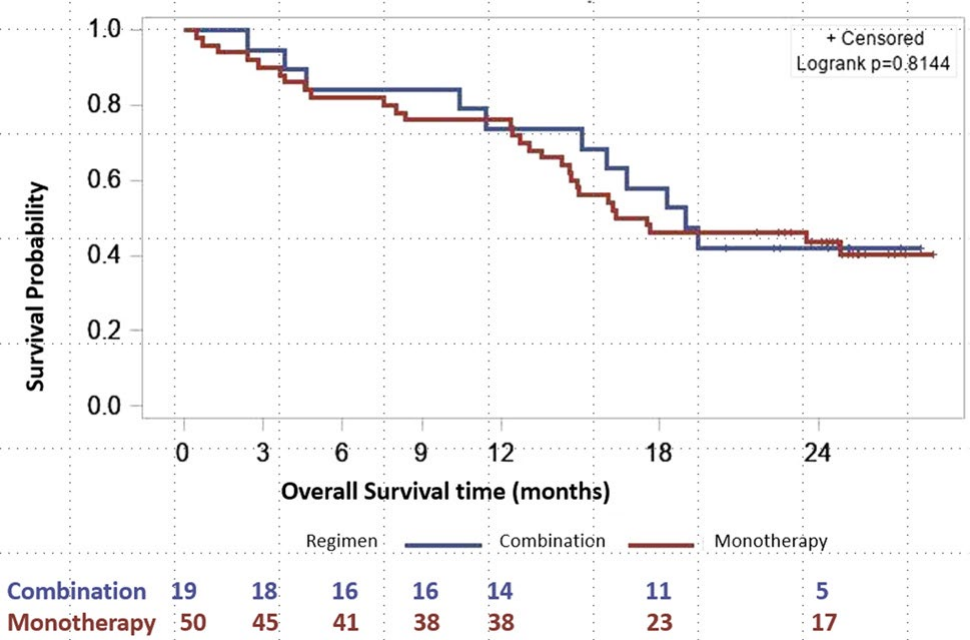
Overall survival according to regimen received (abemaciclib as monotherapy or in combination)

**Fig. 4:**
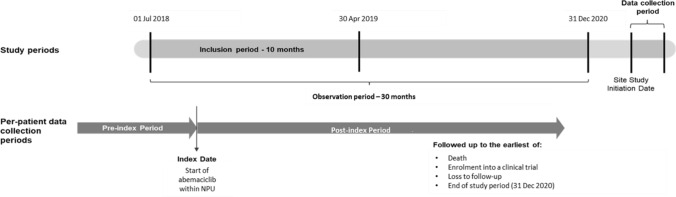
The AbemusS study design. *NPU* Named Patient Use Program

The PFS at 6 months was 54.0% for patients receiving abemaciclib monotherapy and 73.7% for those receiving combination therapy, with a PFS at the end of follow up of 14.0% and 15.8%, respectively. Median PFS was 7.3 months in patients receiving abemaciclib monotherapy and 13.0 months in those receiving combination therapy (Fig. 2). OS at six months was 82.0% and 84.2% for monotherapy and combination therapy, respectively with 42.0% and 42.1% of patients alive at this point in time. Median OS was 16.9 months for patients receiving monotherapy, and 19.0 months for those receiving combination therapy (Fig. 3). For TTP, patients were censored if they died or did not progress, and median TTP was 8.5 months in the monotherapy group and 13 months in the combination therapy group.

## Discussion

To our knowledge, our study is the first observational study based on an NPU Program with abemaciclib in a European population, in near real-world use. AbemusS gathered data for 70.4% of HR+/HER2− mBC patients who received abemaciclib within the Spain NPU Program (69 of 98 patients). A major strength of the study is the broad inclusion criteria of the NPU Program and the long follow-up period of 23 months.

The AbemusS study population had more advanced disease and worse prognosis than patients in abemaciclib mBC clinical trials. Both treatment groups included patients with an ECOG ≥ 2, nine (24.3%) in the monotherapy group and one (7.7%) in the combination therapy group. In contrast, both MONARCH-1 [14] and MONARCH-2 [11] clinical trials only included patients with ECOG 0/1 [7, 11, 14].

Compared with the abemaciclib clinical trials, the AbemusS population had received more prior treatment lines, which was to be expected as the NPU program only included patients who were not candidates for any therapeutic alternative or clinical trials. In the monotherapy group, patients had received a median of 6 lines of prior therapy versus 3 prior lines of treatment in MONARCH-1 [14]. For combination therapy, patients had received a median of 5 lines of prior therapy, whereas in MONARCH-2 [11], patients must not have received more than one ET or any prior chemotherapy.

Treatment patterns also differed between our study and these clinical trials. In the monotherapy group in MONARCH-1 [14], all patients received abemaciclib 200 mg, with three dose reductions allowed, whereas in our study, 52% of patients received abemaciclib 150 mg. In MONARCH-2 [11], all patients received fulvestrant, whereas in AbemusS fulvestrant was part of combination therapy in only 26.3% of patients. The low use of fulvestrant in AbemusS, despite it having the best evidence for us as an adjunct to abemaciclib, may be due to patients having received multiple lines of prior treatments, which likely included fulvestrant.

Even though patients from the NPU who were included in the AbemusS study presented a less favorable prognosis, the clinical effectiveness of abemaciclib was consistent with the clinical efficacy in clinical trials. Specifically, in the AbemusS monotherapy group, 2.3% of patients achieved a CR and 23.3% a PR, with an ORR of 25.6%, median PFS of 7.3 months and OS of 16.9 months. This compares with a 0.0% CR, 19.7% PR, 19.7% ORR, median PFS of 6.0 months and OS of 17.7 months in MONARCH-1 [14]. Similarly, for the AbemusS combination therapy group, 11.8% achieved a CR and 23.5% a PR, with an ORR of 35.5%, median PFS of 13.0 months and OS of 19.0 months, which is comparable to the findings of the MONARCH-2 study, with a CR in 3.5% of patients, a PR in 44.7%, ORR of 48.1% and median PFS of 16.4 months [11].

Furthermore, following treatment with abemaciclib, > 60% of patients who received monotherapy or combination therapy were able to receive additional treatment, likely representing good tolerability, allowing patients to undergo further treatment when required.

Our study population is likely to differ from a real-world setting, as only those with no other treatment options were included in the NPU, with such patients likely to have progressed further along the path of treatment options, be less likely to respond to treatment, and be at a higher risk of adverse events, which could limit the generalizability of our findings. The main limitations of this study are those inherent to its retrospective design, such as missing values of certain variables and potential inconsistencies or mistakes in available information in the medical records. It should also be noted that the sample size was only 69 patients, and therefore the findings should be interpreted with caution. Additionally, the use of abemaciclib as monotherapy is not in line with the EMA approval of abemaciclib [10]. Finally, the study did not collect safety information, which can be an important factor in treatment decisions.

In conclusion, to our knowledge, this study has the longest follow up of any abemaciclib observational study, and is the first one, outside of the clinical trial setting, to obtain OS data with abemaciclib [18, 19]. Baseline characteristics of women treated within the NPU program suggest an advanced disease course and worse prognosis than in patients enrolled in abemaciclib clinical trials, with a heavily pre-treated population, a high tumor load, and a high proportion of patients with bone and visceral disease. Despite baseline differences, our results suggest that abemaciclib effectiveness both as monotherapy and as part of combination therapy, is consistent with the findings of previous abemaciclib trials.

## Data Availability

The data that support the findings of this study are not openly available due to reasons of sensitivity and are available from the corresponding author upon reasonable request.

## Appendix

See Fig. 4.
